# Further Characterization of the Pseudo-Symmetrical Ribosomal Region

**DOI:** 10.3390/life10090201

**Published:** 2020-09-14

**Authors:** Mario Rivas, George E. Fox

**Affiliations:** Department of Biology & Biochemistry, University of Houston, Houston, TX 77204, USA; mrivasme@central.uh.edu

**Keywords:** origin of life, peptidyl-transferase center, pseudo-symmetry, proto-ribosome, SymR, emergence of biological systems, RNA ligation, dimerization

## Abstract

The peptidyl transferase center of the modern ribosome has been found to encompass an area of twofold pseudosymmetry (SymR). This observation strongly suggests that the very core of the ribosome arose from a dimerization event between two modest-sized RNAs. It was previously shown that at least four non-standard interactions exist between the two halves of SymR. Herein, we verify that the structure of the SymR is highly conserved with respect to both ribosome transition state and phylogenetic diversity. These comparisons also reveal two additional sites of interaction between the two halves of SymR and refine our understanding of the previously known interactions. In addition, the possible role that magnesium may have in the coordination, stabilization, association, and evolutionary history of the two halves (A-region and P-region) was examined. Together, the results identify a likely site where structural elements and Mg^2+^ ions may have facilitated the ligation of two aboriginal RNAs into a single unit.

## 1. Introduction

Ribosomes are responsible for the coded synthesis of proteins one residue at a time in all living systems. Atomic resolution structures have revolutionized our understanding of the ribosome. Peptide bond synthesis occurs in the peptidyl transferase center (PTC), of the large ribosomal subunit, which is comprised exclusively of RNA [1,2]. The synthesis itself is facilitated by proper positioning of substrates [3]. In order to make room for the next synthesis cycle, the growing peptide enters an RNA cavity or pore [4,5] that over evolutionary time has grown to be the exit tunnel, from which the product peptide emerges [6,7,8]. The most widely accepted hypothesis on the evolution of the ribosome states that the most ancient part of it is represented by the PTC and the ribonucleotides that delineate it [9,10,11,12,13,14]. Alternative views suggest that components of the small subunit evolved earlier than the PTC [15] or that the rRNA evolved by accretion from preexisting tRNA-like molecules [16].

An important advancement was presented at the 28th FEBS meeting in 2002 [3] when it was first reported that there was an area of twofold pseudo-symmetry (SymR) in and around the PTC which is comprised of ≈180 RNA residues. SymR has two halves, one associated with the ribosomal A site and the other with the P site [17]. It has been suggested to be the equivalent of the proto-ribosome, the ancestor of the current ribosome [18,19]. SymR is thought to have come into existence by a heterodimerization event in the prebiotic world. Recent studies have pointed out the possible role of tRNA fragments in this and similar events in the prebiotic world [20,21]. In order to facilitate dimerization, the two separate halves would have to have been able to associate. Following dimerization, the resulting SymR would likely utilize RNA/RNA interactions to form a stable structure. A number of potential standard base pairs exist within each half, but none of these connect the two halves. Thus, the interaction between the two halves must involve non-standard interactions.

An early examination of atomic resolution ribosome structures from the bacterium *Deinococcus radiodurans*, the archaea *Haloarcula marismortui*, and the low-resolution structure of the thermophile *Thermus thermophilus* was undertaken [17,18]. This comparison allowed the authors to conclude that the structure of the SymR was in fact likely stabilized by four non-standard interactions between the two halves [17]. Subsequently, SymR was experimentally examined using the quantum kernel energy method [22]. This study confirmed that the full SymR was in fact likely to form a stable structure when alone or when interacting with substrates.

Although it was not explored in earlier studies, another likely stabilizing element is metal ions. Mg^2+^ ions, in particular, are well-known to be essential for the folding and stability of large RNAs that form compact structures, such as the ribosome [23]. On their own, Mg^2+^ ions seem to be crucial for ribosome stabilization and function [24]. Although less frequent, Mg^2+^ ions also coordinate RNA/protein interactions, such as the association of ribosomal protein uL2 with the 23S rRNA [25].

In the present study, we present evidence for the persistence of SymR and its RNA/RNA interactions with respect to both ribosome transition states and phylogenetic diversity. The possible role that some of these interactions and the magnesium ion contacts may have in the stabilization of the A-region and P-region is also discussed. The results suggest a region where RNA/RNA interactions and Mg^2+^ ion coordination may have facilitated the ligation of two aboriginal RNAs.

## 2. Materials and Methods

The pseudo-symmetric region (SymR) proposed by Agmon and coworkers [17] was superimposed over various ribosomal large subunit structures using the cealign algorithm that is integrated into PyMOL (The PyMOL Molecular Graphics System, Version 2.3.3, Schrödinger, New York, NY, USA). This allowed identification of equivalent SymR positions in each structure. SymR structures were initially obtained from crystallographic structures of the ribosome’s large subunit from *Escherichia coli* (*E. coli*) and *Thermus thermophilus (T. thermophilus)*. These structures capture the ribosomes in four transition states: classical PRE translocation (PDB ID 4V9D, 4WOI, 3JBU, 4WPO, and 5F8K), chimeric hybrid (PDB ID 4V7B, 4W29 and 4V9L), hybrid (PDB ID 4V9D, 4WOI, 4V9H, and 4V90), and classical POST translocation (PDB ID 3JCD, 4V67, and 4V51). Next, each corresponding SymR structure was analyzed using the Dissecting the Spatial Structure of RNA (DSSR software, v1.8.9) [26] to search for common structural features with the purpose of identifying those features that may vary as the ribosome transitions across each state. The exact position of the bases that form SymR in the context of the secondary structure of the large subunit (LSU) is shown in Figures S1 and S2.

The same procedures were repeated using crystallographic structures of the ribosome’s large subunit from distantly related organisms belonging to the three domains of life [27] to obtain the corresponding SymR ribonucleotides. In particular, the crystallographic structures, (PDB IDs 1NKW and 1JJ2 1NJP, 1VQN, 1FFK, and 4V4Q) that were originally utilized by Agmon [17,18] to define SymR were revisited. These structures belong to *Deinococcus radiodurans (D. radiodurans)*, *Haloarcula marismortui (H. marismortui),* and *E. coli*. Other *D. radiodurans*, *Methanocaldococcus jannaschii (M. jannaschii)*, *Pyrococcus furiosus (P. furiosus)*, *Saccharomyces cerevisiae (S. cerevisiae),* and *Homo sapiens (H. sapiens)* crystallographic structures (PDB IDs: 5DM7, 4V4N, 4V6U, 4V88, and 6EK0), as well as previous SymR structures from *E. coli* and *T. thermophilus* in classical PRE and hybrid transition states (PDB IDs 4V9D, 4WOI, 3JBU, 4WPO, 5F8K, 4V9H, and 4V90) were also included in this analysis.

All corresponding SymR structures were analyzed using the DSSR software v1.8.9 [26] in the search for common elements that would indicate universal A- to P-region interactions. The DSSR software incorporates the Leontis-Westhof classification of base pairs [28] to identify non-standard base–base interactions. Phylogenetic conservation of bases described in the text was based on the RiboVision suite [29]. Visual inspection over all SymR structures was also performed to avoid discarding possible interactions that might be ignored by the DSSR software due to threshold values, local low-resolution elements, or nonstandard predefined interactions. Visualization of structures and distance measurements of potentially relevant hydrogen bonds was performed over each SymR crystallographic structure with the PyMOL software, and superimpositions were done with the cealign algorithm that is integrated into PyMOL.

Analysis of the conservation of magnesium ions associated with SymR was facilitated by using the Mg^2+^ ions catalogue proposed by Klein and coworkers [23]. This catalogue was originally generated using the *H. marismortui* large subunit crystallographic structure (PDB ID 1S72). Then, the original SymR [17] alone was superimposed over this *H. marismortui* LSU structure to obtain the equivalent ribonucleotides involved in the SymR. Mg^2+^ ions that have contact with at least one ribonucleotide within the *H. marismortui* SymR were selected for analysis. Next, SymR from *H. marismortui* was superimposed over crystallographic structures from phylogenetically distant organisms for which Mg^2+^ ion data was reported and included within each crystallographic structure. This included a different structure from *H. marismortui* (PDB ID 1JJ2), as well as crystal structures from *D. radiodurans* (PDB ID 5DM7), *T. thermophilus* (PDB ID 4WPO), *E. coli* (PDB ID 4V9D), and *S. cerevisiae* (PDB ID 4V88). The LSUs from these structures were aligned against the SymR derived from *H. marismortui* (PDB ID 1S72) using the cealign algorithm that is integrated into PyMOL. This allowed identification of SymR ribonucleotides that may coordinate equivalent Mg^2+^ ions within their respective crystallographic structures. Then, each SymR was displayed with its respective Mg^2+^ data over it to corroborate whether these contacts were in fact conserved on each structure.

## 3. Results

### 3.1. On the Stability of the Structural Features of SymR During Translation

SymR seems to be a stable structure that is well-conserved at the core of the LSU [17]. It is comprised, on average, of 178 nucleotides that form six helices, 13 stems, and eight single-stranded segments. In order to fully assess the extent to which the structure of SymR is conserved during protein synthesis, it was initially cut out from the large subunit crystallographic structures of *E. coli* and *T. thermophilus*. These structures represented four different transition states of the ribosome, including classical PRE translocation, chimeric hybrid, hybrid, and the classical POST translocation state, as defined by Mohan and Noller [30]. Detailed examination using the DSSR software [26] allowed identification of structural elements that are consistently seen as the ribosome transitions between the four states. SymR remains a consistent structure from one state to the next. Minor changes are seen that can be attributed to an intrinsic capacity of its phosphorous backbone to absorb tension. There are also some changes in the orientation of sugar and bases, such as bases that are splayed apart. The latter may result in changes at the nucleotide association level, which immediately impact the number of base pairs, the association of nucleotides into multiplets, and even the formation of long-range interactions, like the formation or dissociation of an extended A-minor. Nevertheless, the impact on the main elements of the structure, such as the number of helices, stems, and single-stranded segments appears to be minimal (Table 1). This is despite the fact that some expected variations appeared when comparing structures from different organisms or structures that were solved by distinct high-resolution methods at different resolutions (Tables S1 and S2).

### 3.2. The Conservation of SymR over the Three Domains of Life

In order to assess the conservation of SymR in the three domains of life [27], the corresponding nucleotides were obtained from the large subunit crystallographic structures of phylogenetically distant organisms. These were I) bacteria (*E. coli*, *D. radiodurans,* and *T. thermophilus*), II) archaea (*H. marismortui*, *M. jannaschii,* and *P. furiosus*), and III) eukaryotes (*S. cerevisiae* and *H. sapiens*). This again included crystallographic structures of ribosomes in classic PRE translocation and hybrid states, as previously defined [30]. Using these structures, the structure of SymR [17] was verified. In particular, it was found that most of the A- to P-region stabilizing interactions were conserved among organisms from all three domains of life, as well as between ribosomal states.

Based on a limited sample of crystallographic structures then available, four major points of interaction between the two halves were previously suggested [17], although not fully described. In the present study, the original four points of interaction were verified and two additional regions of nucleotide interaction between the A-region and the P-region were consolidated from previous suggestions [17]. All six regions have now been characterized in detail, as described below.

**Figure 1:**
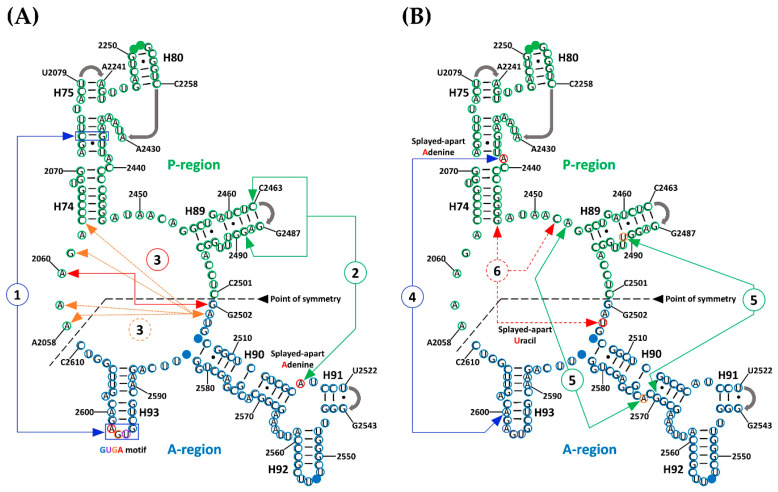
A- to P-region interactions in the symmetric region (SymR) proposed by Agmon [17] pictured over the *T. thermophilus* secondary structure. Bases in the A-region and P-region from the SymR are circled in blue and green, respectively. Bases that interact with the CCA end of the A-site tRNA are marked as solid blue circles (U2506 and G2583 from H90 and G2553 from H92) and bases that interact with the CCA end of the P-site tRNA are marked as solid green circles (G2251 and G2252 from H80). Gray arrows at the end of H75, H80, H89, and H91 represent points of connection that were not detailed by the proponents of the SymR [17]. Six highly conserved regions of long-range RNA interactions have been identified either by the DSSR software or by visual inspection. In order to avoid confusion, the first three of these interactions are illustrated on Panel (**A**) (1, 2, and 3), and the other three are illustrated on Panel (**B**) (4, 5, and 6). Splayed-apart A2519, A2439, U2491, and U2504 are circled in red. As described in the text, the third and fifth interactions both consist of two elements.

**Figure A1:**
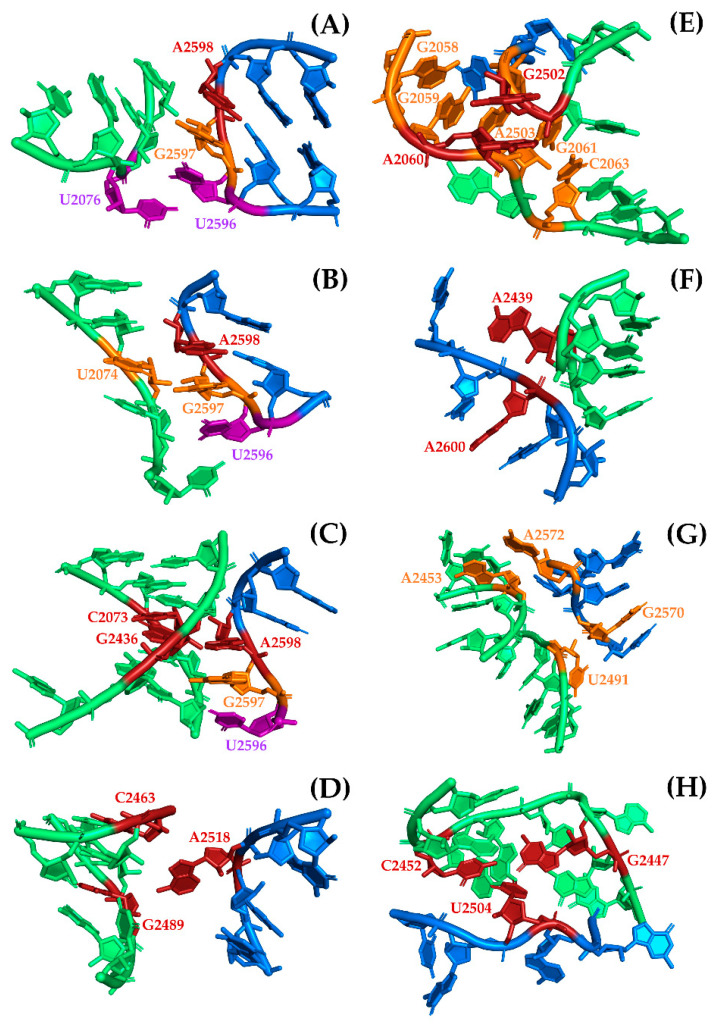
Close view of the A- to P-region interactions that contribute to the association and stabilization of SymR. Bases that are not directly involved in interactions are visualized in cartoon mode and colored blue and green to denote that they belong to either the A-region or the P-region respectively (same color code as Figure 1). (**A**) U2596 (purple) from the GUGA motif base stacks with G2597 (orange), A2598 (red) and U2076 (purple). (**B**) G2597 (orange) from the same motif base pairs with U2074 (orange). (**C**) A2598 (red) from the GUGA motif creates a type I A-minor with C2073 and G2436 (red). (**D**) Splayed-apart A2518 establishes hydrogen bonds with C2463 and G2489 (red). (**E**) Base-stacking region with two elements, the first involves G2502 and A2060 (red) and the second involves A2503, A2058, A2059, G2061, C2063 and A2577 (orange). (**F**) Splayed apart A2439 that created hydrogen bond with A2600 (red). (**G**) Two H89 to H90 contacts represented by A2572 and A2453, and U2491 and G2570 (orange). (**H**) Splayed-apart U2504 that base pairs with G2447 and C2452 (red). A detailed description of each interaction is provided in the text.

The first region (Figure 1A, Figure A1, and Table S3) involves multiple interactions between a GNRA tetraloop motif [31] that is formed at the tip of H93 and the helical region of H74. This motif was previously reported as mediating RNA tertiary interactions, thereby stabilizing the proposed symmetrical dimers [17,18]. It is represented by the almost universal sequence GUGA from H93 that is conserved in all the structures that were used in the analysis. Of the four bases, the triplet UGA represents those nucleotides that are splayed-apart from H93 (A-region) and are exposed into the solvent, thereby allowing interaction with the accepting helical section of H74 from the P-region. Because of its position with respect to H74, U2596 of the triplet is mainly involved in base-stacking with succeeding bases G2597, A2598, and in some cases with U2076. The latter is a non-paired base located at the intersection of H74 and H75. The second base of the triplet, G2597, established a non-standard cSS (Sugar-Edge/Sugar-Edge Anti-parallel) base pair with U2074 from helix 74, which in some cases, by extension, forms part of a multiplet of four bases into which U2244 and A2435 from H74 are included. The third base of the triplet is a universally conserved adenine (A2598) that sometimes establishes a tSH (Sugar-Edge/Hoogsteen Parallel) base pair with G2595. But by far, the most persistent interaction of this adenine is the creation of a Type I A-minor interaction with a conserved C–G base pair (C2073:G2436) from H74. In addition, it sometimes forms part of a multiplet of five bases in which U2245 from the H75-H80 region is also included.

The second interaction region (Figure 1A, Figure A1, and Table S4) that likely contributes to the stabilization of SymR, as previously suggested [17] is represented by a universally conserved adenine (A2518). Due to its location, within the three-way junction of helices 90, 91, and 92, A2518 is never base-paired and is forced to be splayed apart, thereby enhancing the chances of establishing hydrogen bonds (HBs) with distant parts of the structure. Visual analysis of the SymR structures revealed that A2518 mostly established HBs with the available ribose hydroxyl groups (O2′) of the nearest nucleotides from H89, C2463, and G2489 in the case of *T. thermophilus*. The observed hydrogen bond (HB) distances ranged from moderate (2.5–3.2 Å) to weak (3.2–4.0 Å), according to Jeffrey’s HB classification [32]. In the case of two *D. radiodurans* crystallographic structures (PDB IDs 1NKW and 1NJP), distance measurements seemed to be out of HB range. This can be a discrepancy that arises from crystal resolution issues or because the ribosome crystal was captured in the middle of an association–dissociation event. In any case, there are plenty of structures, even from *D. radiodurans* (PDB ID 5DM7), that showed consistent HB distances suggesting that the interaction of splayed-apart A2518 and helix 89 is beyond doubt (Table S4).

The third region of interaction, (Figure 1A, Figure A1, and Table S5), between the two halves of SymR is divided into two consecutive base-stacking elements. The first is integrated by two bases, the highly conserved splayed-apart G2502 located between H89 and H90 at the beginning of the A-region and the universally conserved splayed-apart A2060 located between H73 and H74. The second stacking element is a more robust one, integrated by the highly conserved, splayed-apart A2503 from the H89–H90 region and A2058, A2059, G2061 from the H73–H74 region, as well as C2063 from H74. This second stacking element varies in the number of bases that are part of it, but it remained constant over all crystallographic structures that were analyzed. For example, the incorporation of A2577 from H90 into the second base-stacking element is a variation that seemed to be exclusive to the *T. thermophilus* crystal structures (Table S5).

Suggested by Agmon [17] and confirmed to be a persistent element by visual inspection of SymR structures, the fourth point of interaction, (Figure 1B, Figure A1, and Table S6), is characterized by another universally conserved splayed-apart adenine (A2439). It is located in the middle of H74. This adenine typically creates a single hydrogen bond that extends from its sugar to the closest phosphate, usually with A2600 (from *T. thermophilus*), which is located in H93. These potential HB distances range from moderate (2.5–3.2 Å) to weak (3.2–4.0 Å). A2439 can also create other HBs with bases or sugars from nucleotides that belong to the H90–H93 region, like U2585 or U2586 from *E. coli* crystal structures. However, these last HBs seemed to vary depending on the crystallographic structure (Table S6).

The fifth interaction region, (Figure 1B, Figure A1, and Table S7), is represented by two hydrogen bonds (HBs) that can be established at two different points where H89 and H90 meet when the structure is completely folded. Distances associated with these HBs again seem to be in a range that goes from moderate (2.5–3.2 Å) to weak (3.2–4.0 Å). The most persistent of these HBs is established between two bases that are not base-paired. The highly conserved splayed-apart A2572 located in H90 established a hydrogen bond (HB) with hydroxyl (O2′) of the ribose from the highly conserved A2453, which is localized in the H74–H89 region. The less persistent of these HBs is created between the sugar of the highly conserved U2491 from H89 and the phosphate of the highly conserved G2570 from H90, although the latter HB seems to vary depending on the crystallographic structure (Table S7).

Finally, the sixth point of interaction (Figure 1B, Figure A1, and Table S8) is characterized by a highly conserved splayed-apart uracil (U2504) from the H89–H90 region that usually creates a cHH (Hoogsteen/Hoogsteen anti-parallel) base pair with the highly conserved G2447 localized at H74 and a cWW (Watson-Crick/Watson-Crick Anti-parallel) base pair with a highly conserved C2452 from the H74–H89 region. Although archaeal crystallographic structures show a different plane of insertion for this uracil, it still creates several hydrogen bonds with other nucleotides in the P-region with distances that also range from moderate to weak (Table S8).

### 3.3. Magnesium Role in Folding and Evolution of the SymR

Initially, Mg^2+^ ion contacts within SymR were assigned to one of three classes based on the bases they stablished contacts with. Class I is characterized by Mg^2+^ ions that are exclusively coordinated by RNA residues that are part of SymR, implying that these interactions could be among the oldest. Class II includes contacts with bases from Domain V outside the SymR. This suggests they arose as the proto-ribosome grew. Class III includes Mg^2+^ ions that contact bases from more distant parts of the LSU RNA, as well as contacts with ribosomal proteins, such as uL2 and uL3 (Table S9). This suggests a more recent acquisition. Conservation of these Mg^2+^ ions over crystal structures from distantly related organisms revealed that the most conserved ions belong to any of these three classes indistinctly (Table 2).

**Figure 2:**
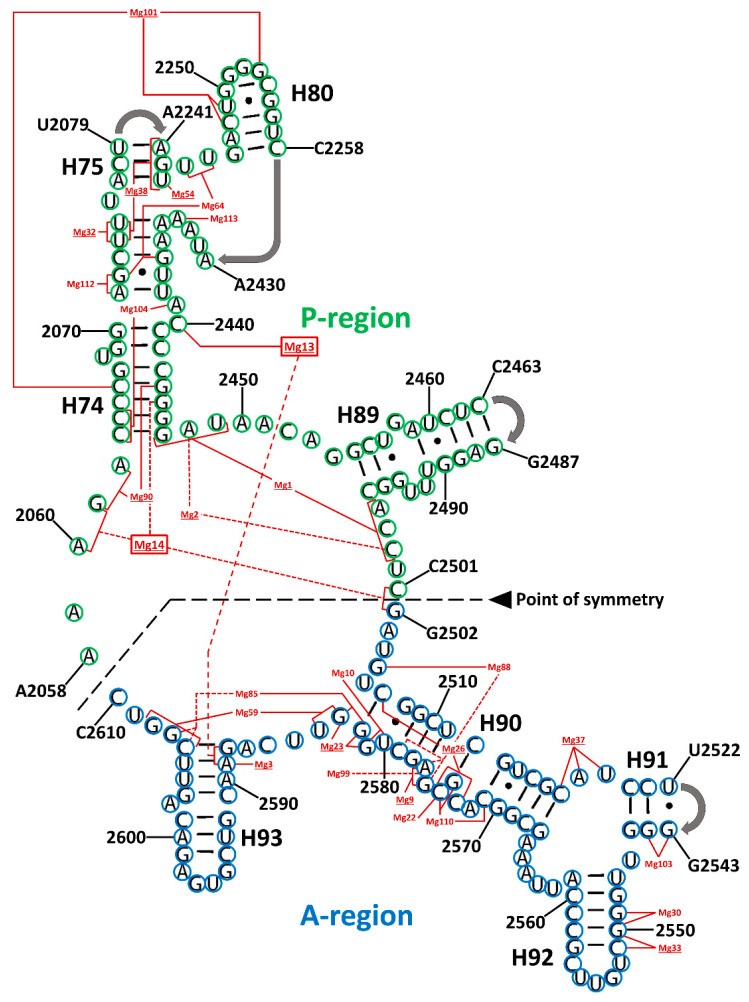
Magnesium ion contacts in the symmetrical region (SymR) modeled over the *T. thermophilus* secondary structure. Bases in the A-region and P-region from the SymR are circled in blue and green, respectively. Mg^2+^ ions are numbered after Klein and coworkers [23]. Underlined Mg^2+^ numbers represent conserved ions that were found in most crystallographic structures (Table S9). Dotted red lines, connecting the bases that coordinate a particular Mg^2+^ ion, were used exclusively to differentiate among lines that crossed with others at some point to avoid confusion. Mg13 and Mg14 are the only metallic ions that established contacts with bases from both the A-region and P-region (red boxes). Full data are presented in Supplementary Tables S9–S11.

As can be appreciated in Figure 2, there are two naturally occurring types of Mg^2+^ ions. The first type facilitates the interaction between distant residues within the SymR secondary structure, thereby assisting in its folding. The second group are coordinated locally by nearby residues, most likely neutralizing electrostatic forces, which is a different mechanism of enhancing the folding.

**Figure 3:**
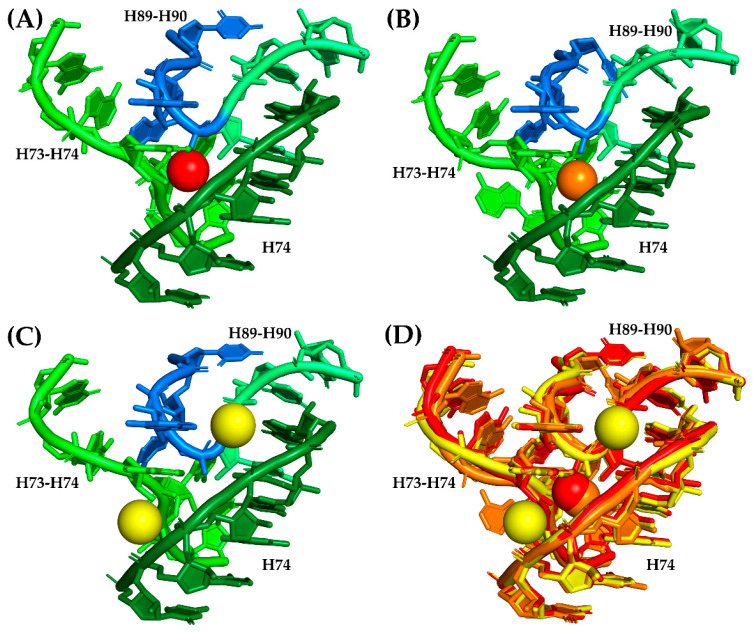
Replacement of conserved Mg14 in *S. cerevisiae*. Mg14 is coordinated in the crystallographic structure from *H. marismortui* (PDB ID 1S72) by A2101, G2102 from the H73–H74 region, G2480 from H74, and C2536 and G2537 from the H89–H90 region, as previously stated by Klein and coworkers [23]. This magnesium ion is conserved among crystallographic structures of *E. coli*, *T. thermophilus*, *H. marismortui,* and *D. radiodurans* (Table S10). (**A**) The coordination region of Mg14 (red sphere) in a different *H. marismortui* structure (PDB ID 1JJ2). Ribonucleotides from the P-region are colored in three tones of green to denote the fact that they belong to distant areas of the P-region, while nucleotides from the A-region are colored in blue. (**B**) Coordination region of Mg14 (orange sphere) in *T. thermophilus* (PDB ID 4WPO). The color code of the bases is the same as in panel A. (**C**) Mg14 coordination region on *S. cerevisiae* (PDB ID 4V88) with two Mg^2+^ ions (yellow spheres, Mg3964 and Mg4328) that seemed to have replaced Mg14 by establishing contacts with the phosphate groups of most of the bases that were supposed to be coordinating Mg14. The color code of the bases is the same as in Panel A. (**D**) Structural alignment of the Mg14 coordination region from *H. marismortui* (red), *T. thermophilus* (orange), and *S. cerevisiae* (yellow) that shows the relative positions of the corresponding Mg^2+^ ions in this region.

A total of 28 magnesium ion contacts were observed within SymR (Figure 2). Of the 28 Mg^2+^ ions contacting SymR nucleotides, only Mg13 and Mg14 established contacts between the A- and P-regions of the SymR (Figure 2, Table 2, and Table S9). However, they are not equally conserved. Of the 28, only 10 are present in all the crystallographic structures of the SymR (Table S10). This last number increases to 16 if we consider those ions that lack an equivalent ion in no more than one structure under analysis (Table 2). The latter speculation rests on the assumption that some of these interactions have an equivalency. For example, in *S. cerevisiae*, Mg14 is absent. However, its coordination has been replaced by the combination of Mg3964 and Mg4328 (Figure 3).

## 4. Discussion and Conclusions

The GUGA motif seems to be among the strongest forces that hold the two RNA pieces together, as previously suggested [17,18]. The two splayed-apart adenines (A2439 and A2518) and the contacts between H89 and H90 were also first observed by Agmon and coworkers [17]. These contacts between H89 and H90 and one splayed-apart adenine (A2518) were reported as features that alternated in different crystal structures [17], most likely due to the lack of available high-resolution structures at that time. We have confirmed that the splayed-apart adenines and the H89 to H90 contacts are indeed consistent features of the SymR that were extracted from extant crystallographic structures. They seem to be among the easiest to form and dissociate, because in most cases, they solely depend on a single HB. We speculate that this feature may have facilitated an early polymerization cycle to take place. Extant ribosomes exhibit regions where tension must be absorbed by its structure, allowing the ribosome to transition from one state to another [3,30]. This may have been the case in the structure of the earliest ribosome as well.

The base-stacking region that forms between two consecutive sets of nucleotides (G2502 and A2060, as well as A2503, A2058, A2059, G2061, and C2063) was previously mentioned as separate interactions that differed in the number of nucleobases that integrate in what we call the second element [17]. The third region of interaction is of special interest because it includes bases in the nearby area surrounding the PTC cavity, that may have served as a scaffold that holds in place the first base of the A-region (G2502). This would facilitate the interaction with the last base of the P-region (C2501). This is similar to what occurs in the active site of many enzymes where a substrate is held before catalysis. In the case of the SymR, it may have eventually resulted in the formation of a covalent bond between the two regions.

Finally, the splayed-apart uracil (U2504) was previously identified as creating a single contact with the ribose of A2572 in the *H. marismortui* crystal structure [17]. This uracil has been observed to be creating non-standard base pairs with a couple of bases over several structures that belong to a considerable number of phylogenetically distant organisms. We speculate that because U2504 is right next to the base-stacking region, it also contributed to the A- to P-region stability and the ligation of both regions.

In summary, the six A- to P- interaction regions are widely conserved in the SymR structures of distantly related organisms from the three domains of life. They also remain present in ribosomes captured in different transition states. This is a good indication of their relevance to the stability of the extant peptidyl transferase center (PTC) and its possible contribution to the assembly of the earliest PTC version, now called the proto-ribosome.

On the other hand, despite discordant visions [15,16] there is a widely accepted hypothesis that assumes that the ribosome is a molecular fossil whose structure recapitulates its history and states that the most ancient part of it is represented by the PTC and the ribonucleotides that delineate it [9,10,11,12,13,14]. By assuming this scenario, we classified Mg^2+^ ions that interact with the SymR in three classes that might say something about the relative time in which these interactions were established. A more classic view, derived from comparative biology, assumes the conservation level over phylogenetically distant organisms as a different way of dating biologically relevant features. With this in mind, we also performed a search of the magnesium ion coordination over several crystallographic structures from distantly related organisms from the three domains of life. The results derived from both approaches do not completely agree on the relative age of the 28 magnesium ions that have contacts with SymR. These contrasting results may reflect the structural dependency of the highly conserved Mg^2+^ ions that has been long-established over evolutionary time within the extant ribosome. Nevertheless, the overlapping results immediately point to relevant Mg^2+^ ion interactions, like the previously described magnesium ion microcluster formed by Mg1 and Mg2 within the P-region (Table 2), that has been recognized as a primeval coordination motif relevant for RNA folding, function, and evolution [24]. Other well-conserved Mg^2+^ ions within classes that suggest early SymR interactions are also of special interest.

If the vision that the proto-ribosome arose by fusion of two similar structures is correct [17,18], then to understand how this occurred is a central question. Based on our results, we also suggest that Mg14 coordination could represent the force that acted over the two pseudo-symmetrical units, thereby facilitating a ligation process between the last nucleotide from the P-region and the first nucleotide from the A-region. This interaction is now represented by C2501 and G2502 (Figure 2 and Figure 3), from which the proto-ribosome could have arisen as a single RNA chain. It would be of value to experimentally determine if two separate RNA fragments would anneal to one another if they exhibit similar magnesium coordination. After all, the ligation of two ribonucleotides aided by magnesium ions has been observed within the extant RNA polymerase II, whose active site coordinates two Mg^2+^ cations that are considered essential for its activity [33].

The results presented here suggest that metallic cations like magnesium might be relevant not only for the folding and catalytic capabilities of the proto-ribosome, but they also play a role in the evolutionary pathway this structure might have followed.

It is well-known that several unsuccessful attempts to synthetize a functional proto-ribosome have been made in the past. The current results may contribute to the understanding of the relevant interactions that must be observed if a proto-ribosome is expected to exhibit structural integrity and catalytic function. Moreover, the well-established requirement of magnesium should not be overlooked if experimental approaches are to be taken, at least during early stages.

## Figures and Tables

**Table 1:** Summary of the structural elements detected by DSSR software on SymR derived from eight *T. thermophilus* LSU crystallographic structures captured in four transition states. Base–base interactions include standard and non-standard base pairs. Each SymR structure is comprised of 178 nucleotides.

Transition State	Classic PRE		Chimeric Hybrid		Hybrid		Classical POST	
PDB ID (Chain)	4WPO (CA)	5F8K (2A)	4W29 (BA)	4V9L (BA)	4V9H (BA)	4V90 (BA)	4V67 (BA)	4V51 (BA)
Base-Base interactions	89	86	85	79	86	87	83	85
Multiplets	17	15	13	14	14	16	15	14
Helices	6	6	6	6	6	6	6	6
Stems	13	13	13	13	13	13	13	13
Isolated WC/wobble pairs	5	6	5	7	5	5	5	5
Atom-base-capping interactions	14	13	13	14	12	11	10	15
Splayed-apart dinucleotides	33	31	33	32	36	34	31	35
Hairpin loops	3	3	3	3	3	3	3	3
Bulges	7	8	7	8	8	8	8	8
Internal loops	3	3	3	4	2	2	2	2
Junctions	1	1	1	1	1	1	1	1
Non-loop single-stranded segments	8	8	8	8	8	8	8	8
A-minor (types I and II) motifs	4	4	4	4	4	4	4	4
eXtended A-minor (type X) motifs	1	1	0	1	1	1	0	0
Ribose zippers	3	3	2	3	3	3	3	3

**Table 2:** Classification of the 16 highly conserved magnesium ions that contact SymR. These were derived from comparisons of crystallographic structures from phylogenetically distant organisms. The numbering system used here was proposed by Klein and coworkers [23]. Magnesium ions that were found present in all structures are represented in bold numbers, while Mg^2+^ ions that were found to be absent only in one crystal structure among those analyzed are represented in italic numbers.

Symmetrical Region Contacts	Mg^2+^ Ion Number	Class
P-region	**1**	I
	*90*	
	**101**	
	**38**	II
	**2**	III
	*32*	
	**54**	
P-region and A-region	*14*	I
	**13**	III
A-region	**23**	I
	**26**	
	*9*	II
	*37*	
	**3**	III
	*30*	
	**33**	

## Supplementary Materials

The following are available online at https://www.mdpi.com/2075-1729/10/9/201/s1, Figure S1: SymR contrasted against the secondary structure of *Thermus thermophilus* ribosome’s large subunit. Figure S2: *Thermus thermophilus* structure (PDB ID 4WPO) of entire large subunit that indicates the area occupied by the SymR and the RNA/RNA interactions within SymR, Table S1: Summary of the structural elements as reported by the DSSR software [26] when applied to SymR derived from crystallographic structures from *E. coli* at four transition states; Table S2: Summary of the structural elements as reported by the DSSR software [26] when applied to SymR derived from crystallographic structures from *T. thermophilus* at four transition states, Table S3: Conserved elements of the GUGA motif from H93 in the A-region that stablished interactions with bases from H74 in the P-region as reported by DSSR software [26], Table S4: Conserved splayed-apart adenine in the H90-H91 zone from A-region that creates hydrogen bonding with bases in H89 from P-region, Table S5: Base-stacking region that is integrated by two consecutive base staking elements as reported by DSSR software [26], Table S6: Conserved splayed-apart adenine in H74 from P-region that creates hydrogen bonding with bases in the H90-H93 zone and H93 from A-region, Table S7: Points of contact between the H90 from A-region and H74-H89 zone and H89 from P-region, Table S8: Conserved splayed-apart uracil (SA-Uracil) from the H89-H90 zone in the A-region that base pair with bases (A-Bases) from H74 and the H74-H89 zone in the P-region, Table S9: Magnesium ion contacts in the symmetrical region that was modeled over the *Haloarcula marismortui* crystallographic structure (PDB ID 1S72), Table S10: Magnesium ion contacts associated to the symmetrical region were verified to persists on other crystallographic structures from *H. marismortui* (PDB ID 1JJ2), *D. radiodurans* (PDB ID 5DM7), *T. thermophilus* (PDB ID 4WPO, Chain CA), *E. coli* (PDB ID 4V9D, Chain DA) and *S. cerevisiae* (PDB ID 4V88, Chain A1), Table S11: Number of the bases that are involved in the Mg^2+^ ion coordination as they appear in the different numbering systems for the *H. marismortui* (PDB ID 1JJ2), *D. radiodurans* (PDB ID 5DM7), *T. thermophilus* (PDB ID 4WPO), *E. coli* (PDB ID 4V9D) and *S. cerevisiae* (PDB ID 4V88) crystallographic structures.

## References

[1] Bashan A., Zarivach R., Schluenzen F., Agmon I., Harms J., Auerbach T., Baram D., Berisio R., Bartels H., Hansen H.A.S. (2003). Ribosomal crystallography: Peptide bond formation and its inhibition. Biopolymers.

[2] Polacek N., Mankin A.S. (2005). The Ribosomal Peptidyl Transferase Center: Structure, Function, Evolution, Inhibition. Crit. Rev. Biochem. Mol. Boil..

[3] Agmon I., Auerbach T., Baram D., Bartels H., Bashan A., Berisio R., Fucini P., Hansen H.A.S., Harms J., Kessler M. (2003). On Peptide Bond Formation, Translocation, Nascent Protein Progression and the Regulatory Properties of Ribosomes: Delivered on 20 October 2002 at the 28th FEBS Meeting in Istanbul. Eur. J. Biochem..

[4] Fox G.E., Tran Q., Yonath A. (2012). An Exit Cavity Was Crucial to the Polymerase Activity of the Early Ribosome. Astrobiology.

[5] Rivas M., Tran Q., E Fox G. (2013). Nanometer scale pores similar in size to the entrance of the ribosomal exit cavity are a common feature of large RNAs. RNA.

[6] Milligan R.A., Unwin P.N.T. (1986). Location of exit channel for nascent protein in 80S ribosome. Nature.

[7] Yonath A., Leonard K.R., Wittmann H.G. (1987). A tunnel in the large ribosomal subunit revealed by three-dimensional image reconstruction. Science.

[8] Voss N.R., Gerstein M., Steitz T., Moore P.B. (2006). The Geometry of the Ribosomal Polypeptide Exit Tunnel. J. Mol. Boil..

[9] Fox G.E., Naik A.K. (2004). The Evolutionary History of the Translations Machinery. The Genetic Code and the Origin of Life.

[10] Hury J., Nagaswamy U., Larios-Sanz M., Fox G.E. (2006). Ribosome origins: The relative age of 23S rRNA domains. Orig. Life Evol. Biosphere.

[11] Fox G.E. (2010). Origin and Evolution of the Ribosome. Cold Spring Harb. Perspect. Boil..

[12] Hsiao C., Mohan S., Kalahar B.K., Williams L.D. (2009). Peeling the Onion: Ribosomes are Ancient Molecular Fossils. Mol. Boil. Evol..

[13] Petrov A.S., Bernier C.R., Hsiao C., Norris A.M., Kovacs N.A., Waterbury C.C., Stepanov V.G., Harvey S.C., Fox G.E., Wartell R.M. (2014). Evolution of the ribosome at atomic resolution. Proc. Natl. Acad. Sci. USA.

[14] Petrov A.S., Gulen B., Norris A.M., Kovacs N.A., Bernier C.R., Lanier K.A., Fox G.E., Harvey S.C., Wartell R.M., Hud N.V. (2015). History of the ribosome and the origin of translation. Proc. Natl. Acad. Sci. USA.

[15] Harish A., Caetano-Anollés G. (2012). Ribosomal History Reveals Origins of Modern Protein Synthesis. PLoS ONE.

[16] Demongeot J., Seligmann H. (2020). Comparisons between small ribosomal RNA and theoretical minimal RNA ring secondary structures confirm phylogenetic and structural accretion histories. Sci. Rep..

[17] Agmon I., Bashan A., Zarivach R., Yonath A. (2005). Symmetry at the active site of the ribosome: Structural and functional implications. Boil. Chem..

[18] Agmon I. (2009). The Dimeric Proto-Ribosome: Structural Details and Possible Implications on the Origin of Life. Int. J. Mol. Sci..

[19] Davidovich C., Belousoff M., Wekselman I., Shapira T., Krupkin M., Zimmerman E., Bashan A., Yonath A. (2010). The Proto-Ribosome: An Ancient Nano-Machine for Peptide Bond Formation. Isr. J. Chem..

[20] Root-Bernstein R., Root-Bernstein M. (2019). The Ribosome as a Missing Link in Prebiotic Evolution III: Over-Representation of tRNA- and rRNA-Like Sequences and Plieofunctionality of Ribosome-Related Molecules Argues for the Evolution of Primitive Genomes from Ribosomal RNA Modules. Int. J. Mol. Sci..

[21] Prosdocimi F., Zamudio G.S., Palacios-Pérez M., De Farias S.T., José M.V. (2020). The Ancient History of Peptidyl Transferase Center Formation as Told by Conservation and Information Analyses. Life.

[22] Huang L., Krupkin M., Bashan A., Yonath A., Massa L. (2013). Protoribosome by quantum kernel energy method. Proc. Natl. Acad. Sci. USA.

[23] Klein D.J., Moore P.B., Steitz T.A. (2004). The contribution of metal ions to the structural stability of the large ribosomal subunit. RNA.

[24] Hsiao C., Williams L.D. (2009). A recurrent magnesium-binding motif provides a framework for the ribosomal peptidyl transferase center. Nucleic Acids Res..

[25] Petrov A.S., Bernier C.R., Hsiao C., Okafor C.D., Tannenbaum E., Stern J., Gaucher E., Schneider D., Hud N.V., Harvey S.C. (2012). RNA–Magnesium–Protein Interactions in Large Ribosomal Subunit. J. Phys. Chem. B.

[26] Lu X.-J., Bussemaker H.J., Olson W.K. (2015). DSSR: An integrated software tool for dissecting the spatial structure of RNA. Nucleic Acids Res..

[27] Woese C.R., Fox G.E. (1977). Phylogenetic structure of the prokaryotic domain: The primary kingdoms. Proc. Natl. Acad. Sci. USA.

[28] Leontis N.B., Westhof E. (2001). Geometric nomenclature and classification of RNA base pairs. RNA.

[29] Bernier C.R., Petrov A.S., Waterbury C.C., Jett J., Li F., Freil L.E., Xiong X., Wang L., Migliozzi B., Hershkovits E. (2014). RiboVision suite for visualization and analysis of ribosomes. Faraday Discuss..

[30] Mohan S., Noller H.F. (2017). Recurring RNA structural motifs underlie the mechanics of L1 stalk movement. Nat. Commun..

[31] Jaeger L., Michel F., Westhof E. (1994). Involvement of a GNRA tetraloop in long-range RNA tertiary interactions. J. Mol. Boil..

[32] Jeffrey G.A. (1997). An Introduction to Hydrogen Bonding.

[33] Svetlov V., Nudler E. (2013). Basic mechanism of transcription by RNA polymerase II. Biochim. Biophys. Acta.

