# Supplementary material for: Further Characterization of the Pseudo-Symmetrical Ribosomal Region
Source: Life (Basel). 2020 Sep 14;10(9):201. doi: 10.3390/life10090201 (PMC7555685; doi:10.3390/life10090201)
Supplement: Supplementary file 1 [file life-10-00201-s001.zip › life-908817-supplementary-proof done/SFigures and STables from Rivas and Fox 2020/SymR Supplemantary Tables.docx]

**Table S1.** Summary of the structural elements as reported by the DSSR software [26] when applied to SymR derived from crystallographic structures from *E. coli* at four transition states. *Electron Microscopy (EM) structures. Total number of base pairs include standard and non-standard base pairs.

| Transition State | Classical PRE | | | Chimeric Hybrid | Hybrid | | Classical POST |
| --- | --- | --- | --- | --- | --- | --- | --- |
| PDB ID (Chain) | 4V9D (DA) | 4WOI (CA) | 3JBU* (b) | 4V7B* (BA) | 4V9D (CA) | 4WOI (BA) | 3JCD* (A) |
| total number of nucleotides | 178 | 178 | 178 | 178 | 178 | 178 | 178 |
| total number of base pairs | 88 | 91 | 85 | 91 | 89 | 90 | 89 |
| total number of multiplets | 15 | 17 | 16 | 16 | 17 | 16 | 14 |
| total number of helices | 6 | 6 | 8 | 6 | 6 | 6 | 8 |
| total number of stems | 14 | 14 | 12 | 14 | 14 | 14 | 13 |
| total number of isolated WC/wobble pairs | 4 | 4 | 6 | 4 | 4 | 4 | 6 |
| total number of atom-base capping interactions | 12 | 12 | 12 | 11 | 14 | 16 | 9 |
| total number of splayed-apart dinucleotides | 31 | 30 | 32 | 31 | 31 | 31 | 32 |
| total number of hairpin loops | 3 | 3 | 3 | 3 | 3 | 3 | 3 |
| total number of bulges | 8 | 8 | 6 | 8 | 8 | 8 | 8 |
| total number of internal loops | 2 | 2 | 4 | 2 | 2 | 2 | 3 |
| total number of junctions | 1 | 1 | 1 | 1 | 1 | 1 | 1 |
| total number of non-loop single-stranded segments | 8 | 8 | 9 | 8 | 8 | 8 | 9 |
| total number of A-minor (types I and II) motifs | 4 | 4 | 3 | 4 | 4 | 4 | 4 |
| total number of eXtended A-minor (type X) motifs | 1 | 0 | 2 | 0 | 0 | 1 | 1 |
| total number of ribose zippers | 3 | 3 | 1 | 3 | 3 | 3 | 3 |

**Table S2.** Summary of the structural elements as reported by the DSSR software [26] when applied to SymR derived from crystallographic structures from *T. thermophilus* at four transition states. *Electron Microscopy (EM) structures. Total number of base pairs include standard and non-standard base pairs.

| Transition State | Classic PRE | | Chimeric Hybrid | | | | Hybrid | | Classic POST | | | |
| --- | --- | --- | --- | --- | --- | --- | --- | --- | --- | --- | --- | --- |
| PDB ID (Chain) | 4WPO (CA) | 5F8K (2A) | 4W29 (BA) | 4W29 (DA) | 4V9L (BA) | 4V9L (DA) | 4V9H (BA) | 4V90 (BA) | 4V67 (BA) | 4V67 (DA) | 4V51 (BA) | 4V51 (DA) |
| total number of nucleotides | 178 | 178 | 178 | 178 | 178 | 178 | 178 | 178 | 178 | 178 | 178 | 178 |
| total number of base pairs | 89 | 86 | 85 | 83 | 79 | 81 | 86 | 87 | 83 | 82 | 85 | 86 |
| total number of multiplets | 17 | 15 | 13 | 11 | 14 | 14 | 14 | 16 | 15 | 14 | 14 | 14 |
| total number of helices | 6 | 6 | 6 | 7 | 6 | 6 | 6 | 6 | 6 | 6 | 6 | 6 |
| total number of stems | 13 | 13 | 13 | 13 | 13 | 13 | 13 | 13 | 13 | 13 | 13 | 13 |
| total number of isolated WC/wobble pairs | 5 | 6 | 5 | 5 | 7 | 5 | 5 | 5 | 5 | 5 | 5 | 5 |
| total number of atom-base capping interactions | 14 | 13 | 13 | 9 | 14 | 11 | 12 | 11 | 10 | 10 | 15 | 12 |
| total number of splayed-apart dinucleotides | 33 | 31 | 33 | 34 | 32 | 31 | 36 | 34 | 31 | 31 | 35 | 35 |
| total number of hairpin loops | 3 | 3 | 3 | 3 | 3 | 3 | 3 | 3 | 3 | 3 | 3 | 3 |
| total number of bulges | 7 | 8 | 7 | 8 | 8 | 8 | 8 | 8 | 8 | 8 | 8 | 8 |
| total number of internal loops | 3 | 3 | 3 | 2 | 4 | 2 | 2 | 2 | 2 | 2 | 2 | 2 |
| total number of junctions | 1 | 1 | 1 | 1 | 1 | 1 | 1 | 1 | 1 | 1 | 1 | 1 |
| total number of non-loop single-stranded segments | 8 | 8 | 8 | 8 | 8 | 8 | 8 | 8 | 8 | 8 | 8 | 8 |
| total number of A-minor (types I and II) motifs | 4 | 4 | 4 | 3 | 4 | 3 | 4 | 4 | 4 | 4 | 4 | 4 |
| total number of eXtended A-minor (type X) motifs | 1 | 1 | 0 | 1 | 1 | 2 | 1 | 1 | 0 | 0 | 0 | 0 |
| total number of ribose zippers | 3 | 3 | 2 | 2 | 3 | 3 | 3 | 3 | 3 | 3 | 3 | 3 |

**Table S3.** Conserved elements of the GUGA motif from H93 in the A-region that stablished interactions with bases from H74 in the P-region as reported by DSSR software [26]. Table shows the crystallographic structures IDs from the PDB that belong to several organisms from Bacteria (*E. coli*, *D. radiodurans* and *T thermophilus*), Archaea (*H. marismortui*, *M. jannaschii* and *P. furiosus*) and Eukaryotes (*S. cerevisiae* and *H. sapiens*) as well as its respective resolution in Angstroms into which the SymR was modeled. Fourth and fifth columns show the uracil on the motif and its corresponding bases that base-stack with it. Sixth and seventh columns show the second guanine in the motif that is paired with one uracil from H74. Eighth and ninth columns show the adenine of the motif and the corresponding base-pair from H74 to which it stablished a type I A-minor interaction. Numbers of the bases correspond to the numbering system that is used within each crystallographic structure. *Electron Microscopy (EM) structures.

| **Organism** | **PDB ID (Chain)** | **Resolution (Å)** | **Uracil** | **Base stacks with** | **Guanine** | **Base pair with** | **Adenine** | **Minor groove**  **base pair** |
| --- | --- | --- | --- | --- | --- | --- | --- | --- |
| *Escherichia coli* | 4V4Q (BB) | 3.6 | U2596 | G2597  A2598  U2076 | G2597 | U2074 | A2598 | C2073:G2436 |
|  | 4V4Q (DB) | 3.6 | U2596 | G2597  A2598  U2076 | G2597 | U2074 | A2598 | C2073:G2436 |
|  | 4V9D (DA) | 3.0 | U2596 | G2597  A2598  U2076 | G2597 | U2074 | A2598 | C2073:G2436 |
|  | 4V9D (CA) | 3.0 | U2596 | G2597  A2598  U2076 | G2597 | U2074 | A2598 | C2073:G2436 |
|  | 4WOI (CA) | 3.0 | U2596 | G2597  A2598 | G2597 | U2074 | A2598 | C2073:G2436 |
|  | 4WOI (BA) | 3.0 | U2596 | G2597  A2598 | G2597 | U2074 | A2598 | C2073:G2436 |
|  | 3JBU* (b) | 3.64 | U2596 | G2597  A2598 | G2597 | U2074 | A2598 | C2073:G2436 |
| *Deinococcus radiodurans* | 1NKW (0) | 3.1 | U2575 | G2576  A2577 | G2576 | U2058 | A2577 | C2056:G2415 |
|  | 1NJP (0) | 3.5 | U2575 | G2576  A2577 | G2576 | U2058 | A2577 | C2056:G2415 |
|  | 5DM7 (X) | 3.0 | U2575 | G2576  A2577  U2059 | G2576 | U2058 | A2577 | C2056:G2415 |
| *Thermus thermophilus* | 4WPO (CA) | 2.8 | U2596 | G2597  A2598  U2076 | G2597 | U2074 | A2598 | C2073:G2436 |
|  | 5F8K (2A) | 2.8 | U2596 | G2597  A2598  U2076 | G2597 | U2074 | A2598 | C2073:G2436 |
|  | 4V9H (BA) | 2.857 | U2596 | G2597  A2598  U2076 | G2597 | U2074 | A2598 | C2073:G2436 |
|  | 4V90 (BA) | 2.95 | U2596 | G2597  A2598  U2076 | G2597 | U2074 | A2598 | C2073:G2436 |
| *Haloarcula marismortui* | 1JJ2 (0) | 2.4 | U2631 | G2632  A2633  U2117 | G2632 | U2115 | A2633 | C2114:G2471 |
|  | 1VQN (0) | 2.4 | U2631 | G2632  A2633  U2117 | G2632 | U2115 | A2633 | C2114:G2471 |
|  | 1FFK (0) | 2.4 | U2631 | G2632  A2633  U2117 | G2632 | U2115 | A2633 | C2114:G2471 |
| *Methanocaldococcus jannaschii* | 4V4N* (A1) | 9.0 | U2711 | G2712  A2713 | G2712 | U2197 | A2713 | C2196:G2551 |
| *Pyrococcus furiosus* | 4V6U* (B1) | 6.6 | U2711 | G2712  A2713 | G2712 | U2197 | A2713 | C2196:G2551 |
| *Saccharomyces cerevisiae* | 4V88 (A1) | 3.0 | U2965 | G2966  A2967 | G2966 | U2416 | A2967 | C2415:G2805 |
|  | 4V88 (A5) | 3.0 | U2965 | G2966  A2967 | G2966 | U2416 | A2967 | C2415:G2805 |
| *Homo sapiens* | 6EKO* (L5) | 2.9 | U4542 | G4543  A4544  G3922 | G4543 | U3920 | A4544 | C3919:G4382 |

**Table S4.** Conserved splayed-apart adenine in the H90-H91 zone from A-region that creates hydrogen bonding with bases in H89 from P-region. Third and fourth columns contain the splayed-apart adenine (SA-Adenine) and the accepting bases (A-Base) to which most likely stablishes hydrogen bonds. Fifth column contains the measured distance of the proposed hydrogen bonds and sixth column contains the elements of the nucleotide that are involved in the hydrogen bonding, P=phosphate, S=sugar and B=base. Measurements extent from the splayed apart adenine to the accepting bases. Numbering system as in table S3. Distances that seem to be out of range for hydrogen bonding are shown in italics and underline. *Electron Microscopy (EM) structures.

| **Organism** | **PDB ID (Chain)** | **SA-Adenine** | **A-Base** | **Distance (Å)** | **HB Extension** |
| --- | --- | --- | --- | --- | --- |
| *Escherichia coli* | 4V4Q (BB) | A2518 | U2489 | 3.1 | B-S |
|  | 4V4Q (DB) | A2518 | U2489 | 3.1 | B-S |
|  | 4V9D (DA) | A2518 | U2489 | 3.3 | B-S |
|  | 4V9D (CA) | A2518 | U2489 | 2.9 | B-S |
|  |  |  | U2491 | 3.4 | B-B |
|  | 4WOI (CA) | A2518 | U2489 | 3.0 | B-S |
|  |  |  | U2491 | 3.5 | B-B |
|  | 4WOI (BA) | A2518 | C2463 | 3.8 | B-S |
|  |  |  | U2489 | 2.9 | B-S |
|  |  |  | U2491 | 3.0 | B-B |
|  | 3JBU* (b) | A2518 | C2463 | 3.7 | B-S |
|  |  |  |  | 3.9 | B-S |
| *Deinococcus radiodurans* | 1NKW (0) | A2497 | U2441 | *4.8* | B-S |
|  |  |  | U2470 | *4.6* | B-B |
|  | 1NJP (0) | A2497 | G2468 | *4.8* | B-S |
|  |  |  | U2470 | *4.7* | B-B |
|  | 5DM7 (X) | A2497 | C2442 | 3.7 | B-S |
|  |  |  | G2468 | 2.7 | B-S |
|  |  |  | U2470 | 3.8 | B-B |
| *Thermus thermophilus* | 4WPO (CA) | A2518 | C2463 | 3.5 | B-S |
|  |  |  |  | 3.8 | B-S |
|  |  |  | G2489 | 3.2 | B-S |
|  | 5F8K (2A) | A2518 | C2463 | 3.6 | B-S |
|  |  |  |  | 3.9 | B-S |
|  |  |  | G2489 | 3.1 | B-S |
|  | 4V9H (BA) | A2518 | C2463 | 3.9 | B-S |
|  |  |  | G2489 | 2.8 | B-S |
|  | 4V90 (BA) | A2518 | C2463 | 4.0 | B-S |
|  |  |  | G2489 | 3.1 | B-S |
| *Haloarcula marismortui* | 1JJ2 (0) | A2553 | C2498 | 3.8 | B-S |
|  |  |  | G2524 | 3.0 | B-S |
|  | 1VQN (0) | A2553 | C2498 | 3.7 | B-S |
|  |  |  | G2524 | 3.0 | B-S |
|  | 1FFK (0) | A2553 | C2498 | 3.9 | B-S |
|  |  |  | G2524 | 3.1 | B-S |
| *Methanocaldococcus jannaschii* | 4V4N* (A1) | A2633 | C2578 | 3.6 | B-S |
|  |  |  |  | 3.8 | B-S |
|  |  |  | G2604 | 3.5 | B-S |
|  |  |  | C2606 | 4.0 | B-B |
| *Pyrococcus furiosus* | 4V6U* (B1) | A2633 | C2578 | 3.3 | B-S |
|  |  |  |  | 3.6 | B-S |
|  |  |  | G2604 | 3.0 | B-S |
|  |  |  | C2606 | 3.7 | B-B |
| *Saccharomyces cerevisiae* | 4V88 (A1) | A2887 | C2832 | 3.9 | B-S |
|  |  |  | U2858 | 3.1 | B-S |
|  | 4V88 (A5) | A2887 | C2832 | 3.8 | B-S |
|  |  |  | U2858 | 3.2 | B-S |
| *Homo sapiens* | 6EKO* (L5) | A4464 | U4435 | 3.1 | B-S |

**Table S5.** Base stacking region that is integrated by two consecutive base staking elements as reported by DSSR software [26]. The first element of this region is integrated by two splay apart bases, one from A site and one from P site, third and fourth columns. Second element is a much larger stack of bases where one splayed-apart adenine from A-region stacks with other bases from P-region, fifth and sixth columns. Numbering system as in table S3. *Electron Microscopy (EM) structures.

| **Organism** | **PDB ID (Chain)** | **Base Stack 1** | | **Base Stack 2** | |
| --- | --- | --- | --- | --- | --- |
|  |  | **From A-region** | **From P-region** | **From A-region** | **From P-region** |
| ***Escherichia coli*** | 4V4Q (BB) | G2502 | A2060 | A2503 | A2058  A2059  G2061  C2063 |
|  | 4V4Q (DB) | G2502 | A2060 | A2503 | A2058  A2059  G2061  C2063 |
|  | 4V9D (DA) | G2502 | A2060 | A2503 | A2058  A2059  G2061  C2063 |
|  | 4V9D (CA) | G2502 | A2060 | A2503 | A2058  A2059  G2061  C2063 |
|  | 4WOI (CA) | G2502 | A2060 | A2503 | A2058  A2059  G2061  C2063 |
|  | 4WOI (BA) | G2502 | A2060 | A2503 | A2058  A2059  G2061  C2063 |
|  | 3JBU* (b) | G2502 | A2060 | A2503 | A2058  A2059  G2061  C2063  C2064  C2065  C2066  G2067  G2069 |
| *Deinococcus radiodurans* | 1NKW (0) | G2481 | A2043 | A2482 | -- |
|  | 1NJP (0) | G2481 | A2043 | A2482 | G2044  C2046 |
|  | 5DM7 (X) | G2481 | A2043 | A2482 | A2041  A2042  G2044  C2046  C2047 |
| *Thermus thermophilus* | 4WPO (CA) | G2502 | A2060 | A2503 | A2058  A2059  G2061  C2063  A2577** |
|  | 5F8K (2A) | G2502 | A2060 | A2503 | A2058  A2059  G2061  C2063  C2064  A2577** |
|  | 4V9H (BA) | G2502 | A2060 | A2503 | A2058  A2059  G2061  C2063  A2577** |
|  | 4V90 (BA) | G2502 | A2060 | A2503 | A2058  A2059  G2061  C2063  A2577** |
| *Haloarcula marismortui* | 1JJ2 (0) | G2537 | A2101 | A2538 | G2099  A2100  G2102  C2104  C2105 |
|  | 1VQN (0) | G2537 | A2101 | A2538 | G2099  A2100  G2102  C2104  C2105 |
|  | 1FFK (0) | G2537 | A2101 | A2538 | G2099  A2100  G2102  C2104  C2105 |
| *Methanocaldococcus jannaschii* | 4V4N* (A1) | G2617 | A2183 | C2618 | A2182 |
| *Pyrococcus furiosus* | 4V6U* (B1) | G2617 | A2183 | C2618 | A2182 |
| *Saccharomyces cerevisiae* | 4V88 (A1) | G2871 | A2402 | A2872 | A2401  G2403  C2405  C2406 |
|  | 4V88 (A5) | G2871 | A2402 | A2872 | G2403  C2405  C2406 |
| *Homo sapiens* | 6EKO* (L5) | G4448 | A3906 | A4449 | G3904  A3905  G3907  C3909  C3910 |

**Table S6.** Conserved splayed-apart adenine in H74 from P-region that creates hydrogen bonding with bases in the H90-H93 zone and H93 from A-region. Third and fourth columns contain the splayed-apart adenine (SA-Adenine) and the accepting bases (A-Bases) to which it most likely stablished hydrogen bonds. Fifth column contains the measured distance of the proposed hydrogen bonds and sixth column contains the elements of the nucleotide that are involved in the hydrogen bonding, P=phosphate, S=sugar and B=base. Measurements extent from the splayed apart adenine to the accepting bases. Numbering system as in table S3. *Electron Microscopy (EM) structures.

| **Organism** | **PDB ID (Chain)** | **SA-Adenine** | **A-Bases** | **Distance (Å)** | **HB Extension** |
| --- | --- | --- | --- | --- | --- |
| *Escherichia coli* | 4V4Q (BB) | A2439 | U2585 | 3.7 | B-B |
|  |  |  | A2600 | 2.7 | S-P |
|  |  |  |  | 3.6 | S-P |
|  | 4V4Q (DB) | A2439 | U2585 | 3.7 | B-B |
|  |  |  | A2600 | 2.8 | S-P |
|  |  |  |  | 3.5 | S-P |
|  | 4V9D (DA) | A2439 | A2600 | 2.7 | S-P |
|  |  |  |  | 3.4 | S-P |
|  | 4V9D (CA) | A2439 | A2600 | 2.8 | S-P |
|  |  |  |  | 3.6 | S-P |
|  | 4WOI (CA) | A2439 | U2586 | 3.8 | B-S |
|  |  |  | A2600 | 2.9 | S-P |
|  |  |  |  | 3.2 | S-P |
|  | 4WOI (BA) | A2439 | U2585 | 3.9 | B-B |
|  |  |  | U2586 | 3.5 | B-S |
|  |  |  | A2600 | 2.7 | S-P |
|  |  |  |  | 3.1 | S-P |
|  | 3JBU* (b) | A2439 | A2587 | 2.9 | S-P |
| *Deinococcus radiodurans* | 1NKW (0) | A2418 | U2564 | 3.2 | B-P |
|  |  |  | A2566 | 2.6 | S-P |
|  | 1NJP (0) | A2418 | C2565 | 3.3 | S-S |
|  |  |  | A2566 | 3.2 | S-P |
|  |  |  | C2580 | 2.9 | B-S |
|  | 5DM7 (X) | A2418 | A2579 | 3.2 | S-P |
|  |  |  |  | 3.3 | S-P |
| *Thermus thermophilus* | 4WPO (CA) | A2439 | A2600 | 2.9 | S-P |
|  |  |  |  | 3.0 | S-P |
|  | 5F8K (2A) | A2439 | A2600 | 3.1 | S-P |
|  |  |  |  | 3.3 | S-P |
|  | 4V9H (BA) | A2439 | A2600 | 2.8 | S-P |
|  |  |  |  | 3.0 | S-P |
|  | 4V90 (BA) | A2439 | A2600 | 3.0 | S-P |
|  |  |  |  | 3.2 | S-P |
| *Haloarcula marismortui* | 1JJ2 (0) | A2474 | U2621 | 3.5 | B-S |
|  |  |  | A2635 | 3.0 | S-P |
|  | 1VQN (0) | A2474 | U2621 | 3.5 | B-S |
|  |  |  | A2635 | 2.7 | S-P |
|  | 1FFK (0) | A2474 | U2621 | 3.5 | B-S |
|  |  |  | A2635 | 3.0 | S-P |
| *Methanocaldococcus jannaschii* | 4V4N* (A1) | A2554 | A2715 | 3.2 | S-P |
| *Pyrococcus furiosus* | 4V6U* (B1) | A2554 | A2715 | 2.9 | S-P |
| *Saccharomyces cerevisiae* | 4V88 (A1) | A2808 | U2955 | 3.5 | B-S |
|  |  |  | A2969 | 2.5 | S-P |
|  |  |  |  | 3.9 | S-P |
|  | 4V88 (A5) | A2808 | U2955 | 3.7 | B-S |
|  |  |  | A2969 | 2.5 | S-P |
|  |  |  |  | 3.6 | S-P |
| *Homo sapiens* | 6EKO* (L5) | A4385 | A4533 | 3.9 | B-P |
|  |  |  | A4546 | 2.8 | S-P |
|  |  |  |  | 3.7 | S-P |

**Table S7.** Points of contact between the H90 from A-region and H74-H89 zone and H89 from P-region. Third and fourth columns contain the bases which most likely stablished hydrogen bonds designated as first base and second base. Fifth column contains the measured distance of the proposed hydrogen bonds and sixth column contains the elements of the nucleotide that are involved in the hydrogen bonding, P=phosphate, S=sugar and B=base. Measurements extent from the first base to the second base. Numbering system as in table S3. Distances that seem to be out of range for hydrogen bonding are shown in italics and underline. *Electron Microscopy (EM) structures.

| **Organism** | **PDB ID (Chain)** | **First Base** | **Second Base** | **Distance (Å)** | **HB Extension** |
| --- | --- | --- | --- | --- | --- |
| *Escherichia coli* | 4V4Q (BB) | A2572 | A2453 | 3.9 | B-S |
|  | 4V4Q (DB) | A2572 | A2453 | 3.9 | B-S |
|  | 4V9D (DA) | A2572 | A2453 | 3.7 | B-S |
|  | 4V9D (CA) | A2572 | A2453 | 4.0 | B-S |
|  | 4WOI (CA) | A2572 | A2453 | 3.7 | B-S |
|  | 4WOI (BA) | A2572 | A2453 | 3.9 | B-S |
|  | 3JBU* (b) | A2572 | A2453 | *4.6* | B-S |
| *Deinococcus radiodurans* | 1NKW (0) | A2551 | A2432 | 2.5 | S-S |
|  |  | U2470 | G2549 | 2.8 | S-P |
|  | 1NJP (0) | A2551 | A2432 | 2.7 | S-S |
|  |  | U2470 | G2549 | 2.5 | S-P |
|  | 5DM7 (X) | A2551 | A2432 | 3.3 | B-S |
|  |  | U2470 | G2549 | 3.6 | S-P |
| *Thermus thermophilus* | 4WPO (CA) | A2572 | A2453 | 3.5 | B-S |
|  |  | U2491 | G2570 | 2.5 | S-P |
|  | 5F8K (2A) | A2572 | A2453 | 3.2 | B-S |
|  |  | U2491 | G2570 | 2.9 | S-P |
|  | 4V9H (BA) | A2572 | A2453 | 3.5 | B-S |
|  |  | U2491 | G2570 | 2.4 | S-P |
|  | 4V90 (BA) | A2572 | A2453 | 3.7 | B-S |
|  |  | U2491 | G2570 | 3.3 | S-P |
| *Haloarcula marismortui* | 1JJ2 (0) | U2607 | A2488 | 3.8 | B-S |
|  |  | C2526 | G2605 | 3.9 | S-P |
|  | 1VQN (0) | U2607 | A2488 | 3.8 | B-S |
|  |  | C2526 | G2605 | 4.0 | S-P |
|  | 1FFK (0) | U2607 | A2488 | 3.7 | B-S |
|  |  | C2526 | G2605 | 3.7 | S-P |
| *Methanocaldococcus jannaschii* | 4V4N* (A1) | A2687 | A2568 | 3.3 | S-S |
| *Pyrococcus furiosus* | 4V6U* (B1) | A2687 | A2568 | 3.3 | S-S |
|  |  |  | C2688 | 3.9 | S-P |
| *Saccharomyces cerevisiae* | 4V88 (A1) | A2941 | U2822 | 3.8 | B-S |
|  |  | U2860 | G2938 | 3.8 | B-P |
|  |  |  | G2939 | 3.9 | S-P |
|  | 4V88 (A5) | A2941 | U2822 | 3.2 | B-B |
|  |  |  |  | 3.5 | B-S |
|  |  | U2860 | G2938 | 3.2 | B-P |
|  |  |  | G2939 | 3.8 | S-P |
| *Homo sapiens* | 6EKO* (L5) | U4399 | A4518 | 3.6 | B-B |
|  |  |  | C4519 | 3.6 | S-P |
|  |  | U4437 | G451 | 3.7 | S-P |

**Table S8.** Conserved splayed-apart uracil (SA-Uracil) from the H89-H90 zone in the A-region that base pair with bases (A-Bases) from H74 and the H74-H89 zone in the P-region. Third column contains the conserved uracil and fourth column contains the bases to which it base pairs. Fifth column has the type of base pair as reported by DSSR software [26]. When the orientation of the conserved uracil is in a perpendicular plane with respect to the plane of the acceptor bases in *H. marismortui*, *M. jannaschii*, *P. furiosus* and *S. cerevisiae* crystallographic structures it cannot stablish standard base pairing, instead it creates different form of hydrogen bonding. This alternative interaction is reported in the fifth column for these organisms, replacing base pair type with distance values in angstroms and the hydrogen bond extensions elements of the nucleotide that are involved in each hydrogen bond, P=phosphate, S=sugar and B=base. Measurements extent from the uracil to accepting bases. Numbering system as in table S3. *Electron Microscopy (EM) structures.

| **Organism** | **PDB ID (Chain)** | **SA-Uracil** | **A-Bases** | **Type** |
| --- | --- | --- | --- | --- |
| *Escherichia coli* | 4V4Q (BB) | U2504 | G2447 | cHH |
|  |  |  | C2452 | cWW |
|  | 4V4Q (DB) | U2504 | G2447 | cHH |
|  |  |  | C2452 | cWW |
|  | 4V9D (DA) | U2504 | G2447 | cHH |
|  |  |  | C2452 | cWW |
|  | 4V9D (CA) | U2504 | G2447 | cHH |
|  |  |  | C2452 | cWW |
|  | 4WOI (CA) | U2504 | G2447 | cHH |
|  |  |  | C2452 | cWW |
|  | 4WOI (BA) | U2504 | G2447 | cHH |
|  |  |  | C2452 | cWW |
|  | 3JBU* (b) | U2504 | C2452 | cWW |
|  |  |  | U2500 | cWH |
| *Deinococcus radiodurans* | 1NKW (0) | U2483 | G2426 | tWH |
|  |  |  | C2431 | cWW |
|  | 1NJP (0) | U2483 | G2426 | tWH |
|  |  |  | C2431 | cWW |
|  | 5DM7 (X) | U2483 | C2431 | cWW |
| *Thermus thermophilus* | 4WPO (CA) | U2504 | G2447 | cHH |
|  |  |  | C2452 | cWW |
|  | 5F8K (2A) | U2504 | G2447 | cHH |
|  |  |  | C2452 | cWW |
|  | 4V9H (BA) | U2504 | G2447 | cHH |
|  |  |  | C2452 | cWW |
|  | 4V90 (BA) | U2504 | G2447 | cHH |
|  |  |  | C2452 | cWW |
| *Haloarcula marismortui* | 1JJ2 (0) | U2539 | A2488 | 3.1, B-B |
|  |  |  | U2535 | 3.3, B-B |
|  | 1VQN (0) | U2539 | A2488 | 3.9, B-B |
|  |  |  | U2535 | 3.4, B-B |
|  | 1FFK (0) | U2539 | A2488 | 3.1, B-B |
|  |  |  | U2535 | 3.4, B-B |
| *Methanocaldococcus jannaschii* | 4V4N* (A1) | U2619 | A2568 | 3.3, B-B |
|  |  |  | U2615 | 3.7, B-B |
| *Pyrococcus furiosus* | 4V6U* (B1) | U2619 | A2568 | 3.2, B-B |
|  |  |  | U2615 | 3.7, B-B |
| *Saccharomyces cerevisiae* | 4V88 (A1) | U2873 | U2822 | 3.4, B-B |
|  |  |  | U2869 | 3.6, B-B |
|  |  |  |  | 3.3, B-S |
|  | 4V88 (A5) | U2873 | U2822 | 3.1, B-B |
|  |  |  | U2869 | 4.0, B-B |
|  |  |  |  | 3.1, P-S |
|  |  |  |  | 3.0, P-S |
| *Homo sapiens* | 6EKO* (L5) | U4450 | U4399 | cWW |

**Table S9.** Magnesium ion contacts in the symmetrical region that was modeled over the *Haloarcula marismortui* crystallographic structure (PDB ID 1S72). These Mg^2+^ ion contacts represent a subset from the Mg^2+^ ions contacts reported for the entire large ribosomal subunit by Klein and coworkers [23]. Mg^2+^ ion contacts in this subset were distributed in three classes based on the contacts they stablished with ribosomal bases and proteins. Class I is integrated by those Mg^2+^ ions that exclusively established contacts with bases within the SymR. Class II is represented by those Mg^2+^ ions that are described as contacting bases within the SymR and other bases within domain V of the LSU. Class III include those Mg^2+^ ions that stablished contacts with bases from other ribosomal domains apart from domain V and some include contacts with residues from rProteins L2 and L3 (*). Color code represent bases from P-region (green) and A-region (blue), bases from domain V outside symmetric region (dark red) and bases outside domain V and protein residues (black).

| **Symmetry region** | **Class** | **Mg ion Assigned Number** | **Bases Coordinating Mg Ions** |
| --- | --- | --- | --- |
| **P region** | I | 1 | 2483  2533  2534  2482  2484  2532 |
|  |  | 64 | 2277  2278  2471  2113 |
|  |  | 90 | 2103  2102  2479 |
|  |  | 101 | 2281  2282  2107  2286 |
|  |  | 104 | 2104  2105  2474 |
|  |  | 112 | 2112  2113 |
|  | II | 38 | 2115  2116  2271  2274  2275  2276 |
|  | III | 2 | 627  2483  2534  625  626 |
|  |  | 32 | 2115  2116  2272  2273  196-L2*  199-L2* |
|  |  | 54 | 162  2276  163  169 |
|  |  | 113 | 2430  220  2431  2468 |
| **P region**  **&**  **A region** | I | 14 | 2102  2537  2101  2480  2536 |
|  | III | 13 | 877  2623  885  2475  2624  195-L2* |
| **A region** | I | 23 | 2617  2618  2616 |
|  |  | 26 | 2608  2609  2610  2542 |
|  |  | 59 | 2618  2619  2641  2642  2643 |
|  |  | 85 | 2618  2617  2641  2642 |
|  |  | 103 | 2578  2579 |
|  |  | 110 | 2609  2606  2610 |
|  | II | 9 | 2611  2612  2093  2094 |
|  |  | 22 | 2097  2540  2095  2096 |
|  |  | 37 | 2553  2552  2554  2575  2576 |
|  |  | 88 | 2540  2611  2612  2614  2646  2647 |
|  |  | 99 | 2612  2647  2648 |
|  | III | 3 | 876  877  2624  193-L2*  195-L2* |
|  |  | 10 | 836  2615  230-L3*  2616  230-L3* |
|  |  | 30 | 1748  1749  2548  2585 |
|  |  | 33 | 1747  1748  1749  2585  2586 |

**Table S10.** Magnesium ion contacts associated to the symmetrical region were verified to persists on other crystallographic structures from *H. marismortui* (PDB ID 1JJ2), *D. radiodurans* (PDB ID 5DM7), *T. thermophilus* (PDB ID 4WPO, Chain CA), *E. coli* (PDB ID 4V9D, Chain DA) and *S. cerevisiae* (PDB ID 4V88, Chain A1). Column for the *H. marismortui* (PDB ID 1S72) uses the numbering system that was assigned by Klein and coworkers [23]. To denote the existence of an equivalent Mg^2+^ ion within each structure the number of that ion in its respective crystallographic structure was used. Bold and underline numbers represent equivalent ions that were present in all structures while simple underline numbers represent those ions that lack an equivalent ion in no more than one structure that was analyzed.

| **Symmetry region** | ***H. marismortui***  **PDB ID 1S72** | ***H. marismortui***  **PDB ID 1JJ2** | ***D. radiodurans***  **PDB ID 5DM7** | ***T. thermophilus***  **PDB ID 4WPO** | ***E. coli***  **PDB ID 4V9D** | ***S. cerevisiae***  **PDB ID 4V88** |
| --- | --- | --- | --- | --- | --- | --- |
| **P region** | **1** | **8001** | **6067** | **3613** | **3104** | **4239** |
|  | 64 | 8064 | --- | 3486 | --- | 4120 |
|  | 90 | 8090 | 6035 | 3278 | 3064 | --- |
|  | **101** | **8101** | **6140** | **3349** | **3066** | **3949** |
|  | 104 | 8104 | --- | 3457 | --- | --- |
|  | 112 | 8112 | --- | --- | --- | 3942 |
|  | **38** | **8038** | **6053** | **3559** | **3065** | **4028** |
|  | **2** | **8002** | **6032** | **3179** | **3105** | **4265** |
|  | 32 | 8032 | 6022 | 3330 | --- | 3930 |
|  | **54** | **8054** | **6055** | **3549** | **3120** | **3881** |
|  | 113 | 8113 | --- | --- | --- | --- |
| **P region & A region** | **13** | **8013** | **6056** | **3408** | **3024** | **3946** |
|  | 14 | 8014 | 6008 | 3390 | 3063 | --- |
| **A region** | **23** | **8023** | **6047** | **3354** | **3111** | **3848** |
|  | **26** | **8026** | **6019** | **3160** | **3113** | **3925** |
|  | 59 | 8059 | --- | --- | --- | 4294 |
|  | 85 | 8085 | --- | --- | --- | --- |
|  | 103 | 8103 | --- | --- | --- | 4169 |
|  | 110 | 8110 | --- | --- | --- | --- |
|  | 9 | 8009 | 6012 | 3424 | --- | 3943 |
|  | 22 | 8022 | 6016 | --- | --- | 4217 |
|  | 37 | 8037 | 6030 | 3345 | 3072 | --- |
|  | 88 | 8088 | --- | 3362 | --- | 3911 |
|  | 99 | 8099 | --- | --- | --- | --- |
|  | **3** | **8003** | **6051** | **3407** | **3023** | **4031** |
|  | 10 | 8010 | --- | 3383 | --- | 3833 |
|  | 30 | 8030 | --- | 3252 | 3051 | 3934 |
|  | **33** | **8033** | **6021** | **3166** | **3116** | **3915** |

**Table S11.** Number of the bases that are involved in the Mg^2+^ ion coordination as they appear in the different numbering systems for the *H. marismortui* (PDB ID 1JJ2), *D. radiodurans* (PDB ID 5DM7), *T. thermophilus* (PDB ID 4WPO), *E. coli* (PDB ID 4V9D) and *S. cerevisiae* (PDB ID 4V88) crystallographic structures. Magnesium number column uses same system that was used by Klein and coworkers [23].

| **Symmetry region** | **Mg #** | ***H. marismortui*** | ***D. radiodurans*** | ***T. thermophilus*** | ***E. coli*** | ***S. cerevisiae*** |
| --- | --- | --- | --- | --- | --- | --- |
| **P region** | 1 | 2482 | 2426 | 2447 | 2447 | 2816 |
|  |  | 2483 | 2427 | 2448 | 2448 | 2817 |
|  |  | 2484 | 2428 | 2449 | 2449 | 2818 |
|  |  | 2532 | 2476 | 2497 | 2497 | 2866 |
|  |  | 2533 | 2477 | 2498 | 2498 | 2867 |
|  |  | 2534 | 2478 | 2499 | 2499 | 2868 |
|  | 64 | 2113 | 2055 | 2072 | 2072 | 2414 |
|  |  | 2277 | 2223 | 2244 | 2244 | 2612 |
|  |  | 2278 | 2224 | 2245 | 2245 | 2613 |
|  |  | 2471 | 2415 | 2436 | 2436 | 2805 |
|  | 90 | 2102 | 2044 | 2061 | 2061 | 2403 |
|  |  | 2103 | 2045 | 2062 | 2062 | 2404 |
|  |  | 2479 | 2423 | 2444 | 2444 | 2813 |
|  | 101 | 2107 | 2049 | 2066 | 2066 | 2408 |
|  |  | 2281 | 2227 | 2248 | 2248 | 2616 |
|  |  | 2282 | 2228 | 2249 | 2249 | 2617 |
|  |  | 2286 | 2232 | 2253 | 2253 | 2621 |
|  | 104 | 2104 | 2046 | 2063 | 2063 | 2405 |
|  |  | 2105 | 2047 | 2064 | 2064 | 2406 |
|  |  | 2474 | 2418 | 2439 | 2439 | 2808 |
|  | 112 | 2112 | 2054 | 2071 | 2071 | 2413 |
|  |  | 2113 | 2055 | 2072 | 2072 | 2414 |
|  | 38 | 2115 | 2057 | 2074 | 2074 | 2416 |
|  |  | 2116 | 2058 | 2075 | 2075 | 2417 |
|  |  | 2274 | 2220 | 2241 | 2241 | 2609 |
|  |  | 2275 | 2221 | 2242 | 2242 | 2610 |
|  |  | 2276 | 2222 | 2243 | 2243 | 2611 |
|  | 2 | 2483 | 2427 | 2448 | 2448 | 2817 |
|  |  | 2534 | 2478 | 2499 | 2499 | 2868 |
|  | 32 | 2115 | 2057 | 2074 | 2074 | 2416 |
|  |  | 2116 | 2058 | 2075 | 2075 | 2417 |
|  | 54 | 2276 | 2222 | 2243 | 2243 | 2611 |
|  | 113 | 2468 | 2412 | 2433 | 2433 | 2802 |
| **P region**  **and**  **A region** | 13 | 2475 | 2419 | 2440 | 2440 | 2809 |
|  |  | 2623 | 2567 | 2588 | 2588 | 2957 |
|  |  | 2624 | 2568 | 2589 | 2589 | 2958 |
|  | 14 | 2101 | 2043 | 2060 | 2060 | 2402 |
|  |  | 2102 | 2044 | 2061 | 2061 | 2403 |
|  |  | 2480 | 2424 | 2445 | 2445 | 2814 |
|  |  | 2536 | 2480 | 2501 | 2501 | 2870 |
|  |  | 2537 | 2481 | 2502 | 2502 | 2871 |
| **A region** | 23 | 2616 | 2560 | 2581 | 2581 | 2950 |
|  |  | 2617 | 2561 | 2582 | 2582 | 2951 |
|  |  | 2618 | 2562 | 2583 | 2583 | 2952 |
|  | 26 | 2542 | 2486 | 2507 | 2507 | 2876 |
|  |  | 2608 | 2552 | 2573 | 2573 | 2942 |
|  |  | 2609 | 2553 | 2574 | 2574 | 2943 |
|  |  | 2610 | 2554 | 2575 | 2575 | 2944 |
|  | 59 | 2618 | 2562 | 2583 | 2583 | 2952 |
|  |  | 2619 | 2563 | 2584 | 2584 | 2953 |
|  |  | 2641 | 2585 | 2606 | 2606 | 2975 |
|  |  | 2642 | 2586 | 2607 | 2607 | 2976 |
|  |  | 2643 | 2587 | 2608 | 2608 | 2977 |
|  | 85 | 2617 | 2561 | 2582 | 2582 | 2951 |
|  |  | 2618 | 2562 | 2583 | 2583 | 2952 |
|  |  | 2641 | 2585 | 2606 | 2606 | 2975 |
|  |  | 2642 | 2586 | 2607 | 2607 | 2976 |
|  | 103 | 2578 | 2522 | 2543 | 2543 | 2912 |
|  |  | 2579 | 2523 | 2544 | 2544 | 2913 |
|  | 110 | 2606 | 2550 | 2571 | 2571 | 2940 |
|  |  | 2609 | 2553 | 2574 | 2574 | 2943 |
|  |  | 2610 | 2554 | 2575 | 2575 | 2944 |
|  | 9 | 2611 | 2555 | 2576 | 2576 | 2945 |
|  |  | 2612 | 2556 | 2577 | 2577 | 2946 |
|  | 22 | 2540 | 2484 | 2505 | 2505 | 2874 |
|  | 37 | 2552 | 2496 | 2517 | 2517 | 2886 |
|  |  | 2553 | 2497 | 2518 | 2518 | 2887 |
|  |  | 2554 | 2498 | 2519 | 2519 | 2888 |
|  | 88 | 2540 | 2484 | 2505 | 2505 | 2874 |
|  |  | 2611 | 2555 | 2576 | 2576 | 2945 |
|  |  | 2612 | 2556 | 2577 | 2577 | 2946 |
|  |  | 2614 | 2558 | 2579 | 2579 | 2948 |
|  | 99 | 2612 | 2556 | 2577 | 2577 | 2946 |
|  | 3 | 2624 | 2568 | 2589 | 2589 | 2958 |
|  | 10 | 2615 | 2559 | 2580 | 2580 | 2949 |
|  |  | 2616 | 2560 | 2581 | 2581 | 2950 |
|  | 30 | 2584 | 2528 | 2549 | 2549 | 2918 |
|  |  | 2585 | 2529 | 2550 | 2550 | 2919 |
|  | 33 | 2585 | 2529 | 2550 | 2550 | 2919 |
|  |  | 2586 | 2530 | 2551 | 2551 | 2920 |
