# Supplementary material for: Further Characterization of the Pseudo-Symmetrical Ribosomal Region
Source: Life (Basel). 2020 Sep 14;10(9):201. doi: 10.3390/life10090201 (PMC7555685; doi:10.3390/life10090201)
Supplement: Supplementary file 1 [file life-10-00201-s001.zip › life-908817-supplementary-proof done/SFigures and STables from Rivas and Fox 2020/SymR Supplementary Figures.docx]

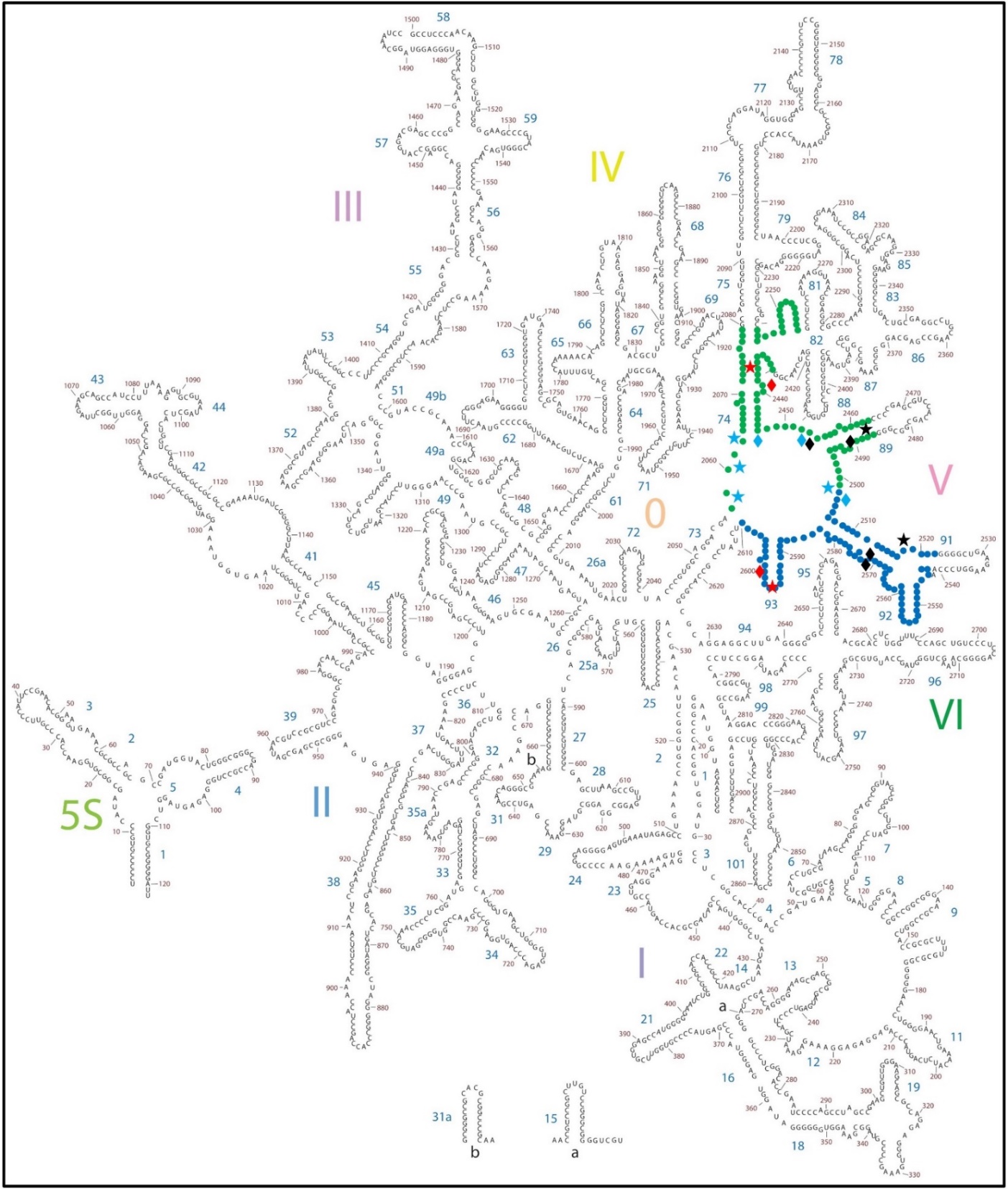


**Figure S1.** Secondary structure of *Thermus thermophilus* ribosome’s large subunit. Ribonucleotides from the SymR [17] are highlighted, nucleotides that belong to the P-region are marked with solid green circles and those from the A-region are marked with solid blue circles. Six points of RNA/RNA interactions are marked. First region is denoted by red stars, second is denoted by black stars, third is marked by light blue stars, fourth region of interaction is marked by red rhombuses, fifth with black rhombuses and sixth is marked with light blue rhombuses. Modified from secondary structures within RiboVision [29].


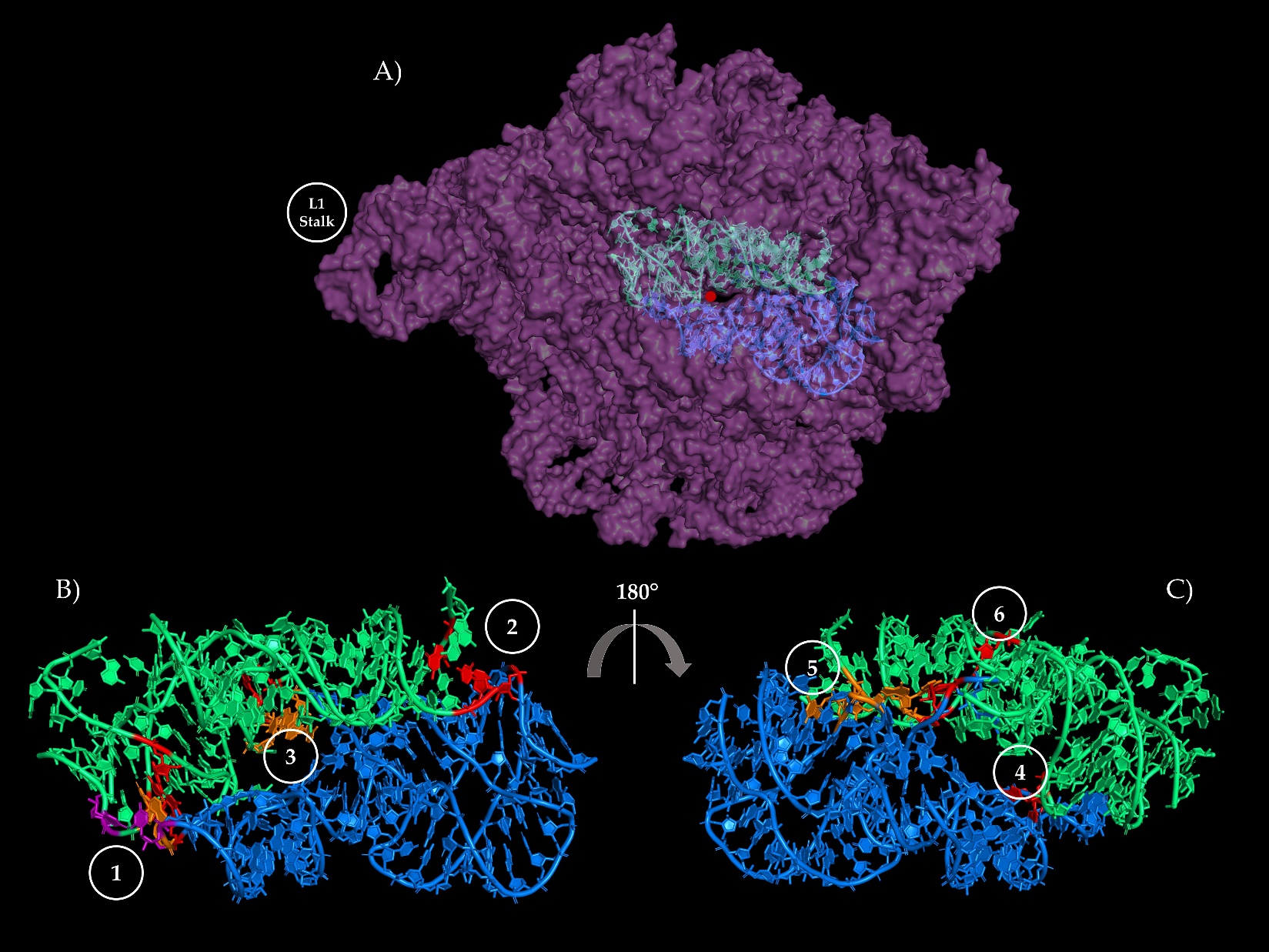


**Figure S2.** *Thermus thermophilus* structure (PDB ID 4WPO) of entire large subunit that indicates the area occupied by the SymR and the RNA/RNA interactions within SymR. A) Crystallographic structure of the SymR with respect to the LSU. Structure is oriented to enable direct view of the PTC cavity or pore [4,5] which is marked with a red dot. The L1 Stalk is also label for orientation reference. B) Closer view of the SymR with the first, second and third RNA/RNA interactions. C) 180° rotation over the Y axis of the close view of the SymR that highlights the fourth, fifth and sixth RNA/RNA interactions. Color code in each panel remains the same as in Figure 1 and Appendix A.
